# Increased Pediatric Head and Neck Abscesses Following the COVID‐19 Pandemic

**DOI:** 10.1002/lary.70742

**Published:** 2026-07-09

**Authors:** Marco DiBlasi, Ryan C. Higgins, Nicole Welch, Dwight Jones, Arthur J. Chang

**Affiliations:** ^1^ Creighton University School of Medicine Omaha Nebraska USA; ^2^ Department of Otolaryngology University of Nebraska Medical Center Omaha Nebraska USA; ^3^ Division of Pediatric Infectious Diseases University of Nebraska Medical Center Omaha Nebraska USA

**Keywords:** abscess, COVID‐19, head and neck, pandemic, pediatric

## Abstract

**Objectives:**

Concerns have emerged that COVID‐19‐related public health measures may have altered pediatric immune development and infection patterns, with studies suggesting an increase in pediatric abscesses following the pandemic. This study evaluated changes in the incidence, clinical features, and management of pediatric head and neck abscesses before, during, and after COVID‐19 lockdown restrictions.

**Methods:**

This retrospective cohort study included 194 patients ≤ 18 years with head and neck abscesses at two tertiary institutions from 2017 to 2024. Cases were identified using ICD‐10 codes and confirmed by chart review. Patients were stratified into pre‐pandemic, lockdown, and post‐pandemic cohorts. Incidence rates were calculated using Poisson regression with pediatric emergency department volume as an offset. Variables were compared using Fisher's exact and Kruskal–Wallis tests. National trends were contextualized using Epic Cosmos data.

**Results:**

Abscess incidence increased from 3.34 to 6.13 to 10.95 per 10,000 emergency department patients per year across the pre‐pandemic, lockdown, and post‐pandemic periods, respectively; representing a 3.28‐fold increase compared to pre‐pandemic levels (*p* < 0.0001). Incidence increased across all anatomic subtypes, including cervical lymphadenitis with abscess, peritonsillar, deep neck space, and intracranial abscesses. Patient demographics, hospital admission rates, and time to treatment did not differ significantly between cohorts. Abscess size was larger during the lockdown period (*p* = 0.047). Post‐pandemic patients more often required repeat surgical intervention (17.4%) compared to pre‐pandemic patients (5.0%).

**Conclusion:**

Pediatric head and neck abscesses increased significantly following the COVID‐19 pandemic, with a sustained rise independent of demographic or treatment‐related factors. Continued surveillance and investigation into post‐pandemic pediatric host susceptibility is warranted.

**Level of Evidence:**

2.

## Graphical abstract

This study evaluated the impact of the COVID‐19 pandemic on pediatric head and neck abscesses at two tertiary care centers. Overall head and neck abscess incidence increased more than threefold in the post‐pandemic period compared with the pre‐pandemic period, with increases observed across all major abscess subtypes. These findings provide one of the first comprehensive US assessments of post‐pandemic trends in pediatric head and neck abscesses.

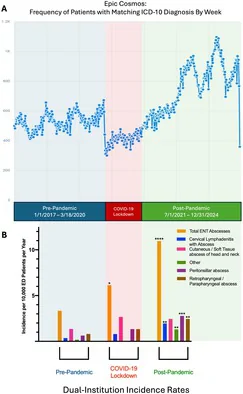

## 1 Introduction

On January 20, 2020, the first US COVID‐19 case prompted executive orders enforcing social distancing, including public school closures [1]. Children were quarantined in their homes, wore masks in public, and experienced markedly reduced social interactions during this lockdown period. These measures persisted until restrictions were gradually lifted in 2021, returning the majority of schools back to in‐person learning [2]. While effectively slowing the spread of COVID‐19, these public health policies are theorized to have reduced children's immune system development due to subsequent limited exposure to common pathogens [3, 4, 5].

After 14 months of pandemic quarantine, the medical community displayed increasing concern that these social distancing measures inadvertently influenced a wave of more severe infections in the pediatric population. In May 2022, the Centers for Disease Control and Prevention detected an increase in pediatric brain abscesses in the United States following the pandemic [6, 7], with surveillance over the subsequent year identifying increased *Streptococcus intermedius* as the major contributing pathogen [8]. The National COVID Cohort Collaborative recently documented the rise of invasive *Streptococcal pyogenes* infections continuing into June 2023 across 58 institutions [9]. However, these trends are not limited to the United States. Two studies from Germany found significant post‐pandemic increases in intracranial infections requiring surgery [10], and acute mastoiditis cases [11]. Furthermore, multiple international studies have similarly documented a significant rise in cervical abscesses in the post‐pandemic era [12, 13, 14, 15, 16, 17]. While these findings support these growing concerns, further rigorous investigation of this trend in the United States remains prudent.

Prior to the pandemic, there was an estimated incidence of up to 0.94 peritonsillar abscess (PTA) cases and 0.22 retropharyngeal abscess cases per 10,000 children in the United States from 2000 to 2009 [18]. These head and neck abscesses are posited to be more prevalent in children due, in part, to their immature immune systems. However, Schweinfurth et al. did not find a correlation between severe head and neck infection patterns and an associated increase in pathogen exposure during school attendance [19], suggesting that age‐related host immune factors, behaviors, and other environmental exposures likely play a role in this increased preponderance for abscess development among pediatric populations. This study aims to further elucidate this possible post‐pandemic increase in pediatric head and neck abscesses at two tertiary care medical institutions before, during, and after the implementation of COVID‐19 lockdown restrictions within the state of Nebraska.

## 2 Materials and Methods

This retrospective cohort study was conducted using all records available at Children's Nebraska and the University of Nebraska Medical Center. The study population included patients aged 18 years or younger who presented between January 1, 2017 and December 31, 2024 with a diagnosis of suppurative cervical adenitis with abscess; peritonsillar or intratonsillar abscess; deep neck space abscess (parapharyngeal, retropharyngeal, prevertebral); intracranial abscess; or cutaneous or soft tissue abscess of the head and neck. Cases were identified using ICD‐10 diagnosis codes (L04.0, L02.01, L02.11, L02.12, J36, J39.0, J39.1, J39.2, M46.22, and G06.0).

All cases underwent chart review, and only those with a clinically or radiographically confirmed abscess as defined above were included. Patients with misdiagnoses or alternative conditions (phlegmon, preauricular pit abscess, Thornwaldt's cyst, lymphadenitis without abscess, post‐transplant lymphoproliferative disorder, tuberculosis, or a non‐tuberculosis mycobacterium infection) were excluded, as were patients with abscesses diagnosed before January 1, 2017 or with incomplete diagnostic or treatment data.

Timeframes were defined as the pre‐pandemic (January 1, 2017 to March 18, 2020; 3.21 years), lockdown (March 19, 2020 to June 30, 2021; 1.28 years), and post‐pandemic (July 1, 2021 to December 31, 2024; 3.50 years) periods. In Nebraska, March 19, 2020 represents the initiation of quarantine protocol for children, and June 30, 2021 represents the day the Public Health Emergency order was lifted, allowing children to return to public schools. Demographic data collected included age at first presentation, sex, race, and ethnicity. Past medical history collected included *Haemophilus influenzae* type B, Pneumococcal, and DTaP/Tdap vaccination status; immunodeficiency status defined by having an immunocompromised disorder diagnosis or being on an immunosuppressing medication (e.g., corticosteroids, IV or subcutaneous immunoglobulins) within 6 months of abscess diagnosis; SARS‐CoV‐2 infection 60 days prior to presentation; and prior head and neck procedures. Objective examination findings upon initial presentation recorded include trismus, decreased neck range of motion, and airway obstruction. The largest recorded abscess diameters (cm) were collected using available imaging upon initial presentation with preference following computed tomography, then magnetic resonance imaging, and then ultrasound. White blood cell count (WBC), absolute neutrophil count (ANC), erythrocyte sedimentation rate (ESR), and C‐reactive protein (CRP) levels upon initial presentation were collected. Aerobic and anaerobic culture results and antibiotic susceptibilities were reviewed and compared to antibiotic selection by a pediatric infectious disease physician. Lastly, treatment data collected included hospital admission status, the dates of symptom onset, antibiotic treatment duration (start date, completed date), and surgical intervention (bedside incision and drainage [I&D] or operative room). Derived outcomes included reported onset of symptoms to last day of antibiotic (total infection duration), to first day of an antibiotic (days until receipt of first antibiotic), to first day of an effective antibiotic (days until receipt of an effective antibiotic), and to the day of surgical intervention (days until first surgical intervention).

Antibiotic courses were consolidated and adjudicated by a pediatric infectious disease physician, with courses less than 48 h or given prior to admission combined into a single course. Antibiotics were classified as empiric or definitive based on culture acquisition and presence of recovered organisms. Appropriateness of antibiotics was determined using available disease‐specific guideline recommendations or comparison of susceptibility from culture to antibiotic selection. In the case of polymicrobial results without specific pathogen identification, antibiotic appropriateness was determined by Infectious Diseases Society of America guidelines [20]. Duration appropriateness was not assessed.

We used Fisher's exact test for categorical data and Kruskal–Wallis test for continuous and ordinal data due to the non‐normal distribution of continuous data. Data was analyzed and graphed using GraphPad Prism 10.6.1. Incidence comparisons were done using a Poisson regression with offset using unique pediatric emergency department (ED) patients seen in each respective time period to account for unequal exposures. Incidence was analyzed in Python 3 using NumPy 1.23.5, Pandas 1.5.3, and SciPy 1.10.0. Post hoc comparisons used included Dunn's multiple comparisons for significant Kruskal–Wallis tests and incidence rate ratios (IRR) with Holm–Bonferroni adjustments. Length of stay (LOS) was calculated from the first hospitalization and/or hospital‐based procedure following the Agency of Healthcare Research and Quality (AHRQ) calculation.

Figure 1A data were derived from Epic Cosmos, a dataset created in collaboration with a community of health systems using Epic, representing 300 million patient records over 1854 hospitals and 42,200 clinics as of December 2025. The community represents patients from all 50 states, District of Columbia, Canada, Lebanon, and Saudi Arabia. The query utilized the same age, date, and ICD‐10 codes used for our query, but due to the deidentified nature of Epic Cosmos data, no further refinement was possible.

**FIGURE 1:**
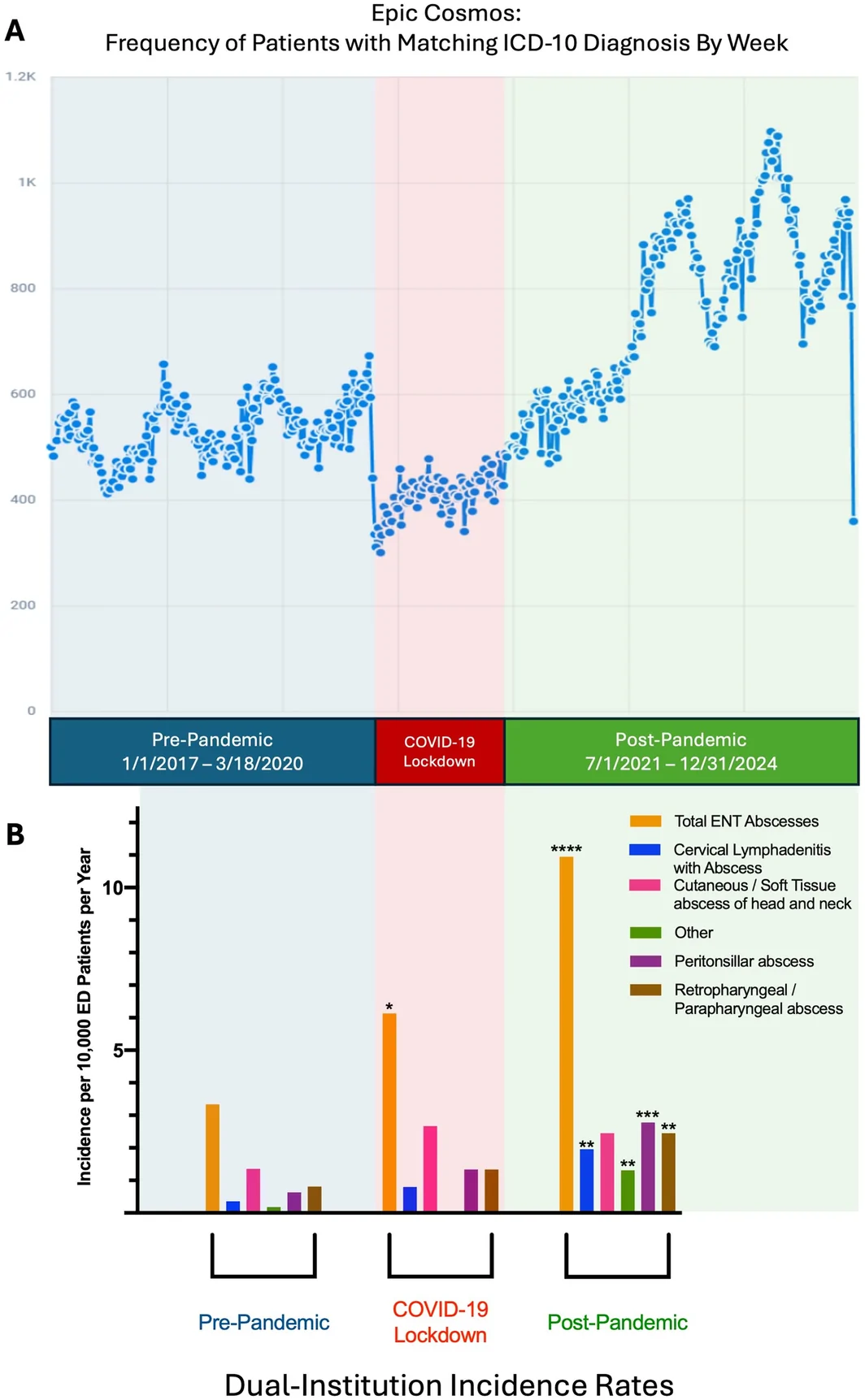
Head and neck abscess frequency and incidence rates. (A) Epic Cosmos query for included ICD‐10 diagnosis codes from January 1, 2017 to December 31, 2024. Frequency rates were stratified on a weekly basis. (B) Incidence rates of head and neck abscesses among the dual institution cohort. Statistical significance was calculated via pairwise incidence rate ratios with Holm–Bonferroni Correction, with the pre‐pandemic patient group as the reference. *p < 0.05; **p < 0.01; and ***p < 0.001, and ****p < 0.0001. [Color figure can be viewed in the online issue, which is available at www.laryngoscope.com]

Chart‐reviewed data were collected and managed using REDCap electronic data capture tools hosted at the University of Nebraska Medical Center [21, 22]. This study received ethics clearance from the University of Nebraska Medical Center Institutional Review Board (Reference Number: 0633‐24‐EP).

## 3 Results

### 3.1 Incidence of Head and Neck Abscesses

Epic COSMOS unadjusted weekly data was populated in Figure 1A. A comprehensive chart review of two institutions was then conducted to further evaluate this trend, but with a refined patient population of true head and neck abscesses.

Initial query captured 508 patients, with final cohort comprised of 194 patients following exclusion criteria. Analysis of the 194 patients included began with assessing the incidence rates of abscesses over the three defined timeframes. In the pre‐pandemic period, there were 37 patients with a head and neck abscess (19.1%) among 110,921 unique ED patients seen during that time period, resulting in an incidence rate of 3.34 abscesses per 10,000 ED patients per year. During the lockdown period, there were 23 patients diagnosed with a head and neck abscess (11.9%) among 37,512 unique ED patients seen during that time period, resulting in an incidence rate of 6.13 abscesses per 10,000 ED patients per year. Lastly, in the post‐pandemic period, there were 134 patients diagnosed with a head and neck abscess (69.1%) among 122,421 unique ED patients seen during that time period, resulting in an incidence rate of 10.95 abscesses per 10,000 ED patients per year. This represented a post‐pandemic period incidence rate 3.28× greater than that of the pre‐pandemic period. Compared to the pre‐pandemic period, the abscess IRR during the lockdown period was 1.838 (95% CI: 1.092–3.093; *p* = 0.022). Compared to the pre‐pandemic period, the abscess IRR for the post‐pandemic period was 3.281 (95% CI: 2.28–4.722; *p* < 0.001). Lastly, compared to the lockdown period, the abscess IRR for the post‐pandemic period was 1.785 (95% CI: 1.147–2.778; *p* = 0.021).

Abscess incidence analysis was then stratified by anatomical location (Tables 1 and 2). The “Other” head and neck abscesses were constituted by 14 intracranial abscesses, 1 laryngeal abscess, 1 intraorbital abscess, and 2 intratonsillar abscesses. Interestingly, there were no “Other” abscesses during the lockdown period (Figure 1B).

**TABLE 1:** Incidence rates of pediatric head and neck abscess subtypes across time periods.

Abscess subtypes incidence rate	Pre‐pandemic (*n* = 37)	Lockdown period (*n* = 23)	Post‐pandemic (*n* = 134)
Suppurative cervical lymphadenitis with abscess	0.36061702	0.799744082	1.960447962
Cutaneous/soft tissue abscess of head and neck	1.35231381	2.665813606	2.450559953
Other	0.18030851	0	1.306965308
Peritonsillar abscess	0.63107978	1.332906803	2.77730128
Retropharyngeal/parapharyngeal abscess	0.81138829	1.332906803	2.450559953

*Note:* Incidence rates are reported as cases per 10,000 pediatric emergency department patients per year for the pre‐pandemic, COVID‐19 lockdown, and post‐pandemic periods. “Other” abscesses include intracranial, laryngeal, intraorbital, and intratonsillar abscesses.

**TABLE 2:** Incidence rate ratios comparing pediatric head and neck abscess subtypes across time periods.

Abscess subtype	Pre‐pandemic vs. lockdown period IRR (95% CI), *p*	Pre‐pandemic vs. post‐pandemic IRR (95% CI), *p*	Lockdown period vs. post‐pandemic IRR (95% CI), *p*
Cervical lymphadenitis with abscess	0.53 (0.12–2.38), *p* = 0.409	0.18 (0.06–0.52), *p* = 0.002	0.34 (0.10–1.13), *p* = 0.079
Cutaneous/soft tissue abscess	0.60 (0.27–1.33), *p* = 0.209	0.55 (0.29–1.01), *p* = 0.055	0.91 (0.45–1.86), *p* = 0.798
Peritonsillar abscess	0.56 (0.18–1.76), *p* = 0.321	0.22 (0.10–0.51), *p* < 0.001	0.40 (0.16–1.03), *p* = 0.057
Retropharyngeal/parapharyngeal abscess	0.72 (0.24–2.14), *p* = 0.554	0.33 (0.16–0.69), *p* = 0.003	0.46 (0.18–1.17), *p* = 0.104
Other	N/A	0.14 (0.03–0.59), *p* = 0.008	N/A

*Note:* Incidence rate ratios (IRRs) with 95% confidence intervals were calculated to compare abscess subtype incidence between the pre‐pandemic, COVID‐19 lockdown, and post‐pandemic periods. *p* values were calculated using Poisson regression models comparing incidence rates across time periods, with pediatric emergency department volume included as an offset. N/A indicates comparisons that could not be calculated due to zero cases in one time period.

### 3.2 Demographics, Past Medical History, and Clinical Presentation

The average age of the cohort was 8.31 years old (SD = 5.96) with the majority being white (74.7%), non‐Hispanic/Latinx (75.3%) males (54.1%). There were no significant differences between the age at diagnosis between pre‐pandemic, lockdown, and post‐pandemic patients (*p* = 0.367). There were no significant differences in patient sex, race, and ethnicity between patients in the pre‐pandemic, lockdown, and the post‐pandemic periods (*p*'s > 0.05) (Table 3).

**TABLE 3:** Demographic frequencies of the entire cohort, stratified over the pre‐pandemic, lockdown period, and post‐pandemic timeframes.

Demographic characteristics	Total (*n* = 194)	Pre‐pandemic (*n* = 37)	Lockdown period (*n* = 23)	Post‐pandemic (*n* = 134)	*p*
Age (mean, SD, years)	8.31 (5.96)	7.36 (5.93)	7.88 (6.72)	8.65 (5.84)	0.3667
Sex (*n*, %)					
Male	105 (54.1%)	22 (59.5%)	9 (39.1%)	74 (55.2%)	0.2779
Female	89 (45.9%)	15 (40.5%)	14 (60.9%)	60 (44.8%)	
Race (*n*, %)					
White	115 (59.3%)	21 (56.8%)	13 (56.5%)	81 (60.4%)	0.2409
Hispanic	30 (15.5%)	8 (21.6%)	3 (13.0%)	19 (14.2%)	
African/African‐American	24 (12.4%)	2 (5.4%)	3 (13.0%)	19 (14.2%)	
Asian American and Pacific Islander	4 (2.15)	0 (0%)	1 (4.3%)	3 (2.2%)	
American Indian	2 (1.0%)	1 (2.7%)	0 (0%)	1 (0.7%)	
Multiracial	12 (6.2%)	1 (2.7%)	2 (8.7%)	9 (6.7%)	
Other	7 (3.6%)	4 (10.8%)	1 (4.3%)	2 (1.5%)	
Ethnicity (*n*, %)					
Non‐Hispanic/Latinx	146 (75.3%)	24 (64.9%)	18 (78.3%)	104 (77.6%)	0.2927
Hispanic/Latinx	48 (24.7%)	13 (35.1%)	5 (21.7%)	30 (22.4%)	

*Note: p* values calculated using Fisher's exact test.

No patients in this study were immunocompromised. 4.3% of lockdown period patients and 3.7% post‐pandemic period patients were diagnosed with SARS‐CoV‐2 within 60 days prior to presentation. There was no significant difference between the pre‐pandemic, lockdown, and post‐pandemic cohorts in regard to having a procedure within six months prior to abscess development. There were no significant differences among pediatric bacterial vaccination rates (Hib, Pneumococcal, and DTaP/Tdap) between the three cohorts. Additionally, hospital admission rates did not differ significantly between pre‐pandemic, lockdown, and post‐pandemic cohorts. Objective examination findings upon initial presentation did not demonstrate any significant associations over the three time periods (Table S1).

The mean abscess diameters were significantly associated for pre‐pandemic, lockdown, and post‐pandemic patients, being 2.4 (SD = 1.24), 3.45 (SD = 1.58), and 2.46 (SD = 1.32), respectively (*p* = 0.047) (Figure 2). However, there were no statistically significant Dunn's pairwise comparisons between time periods regarding abscess size (pre‐pandemic vs. lockdown *p* = 0.0892; pre‐pandemic vs. post‐pandemic *p* > 0.9999; and lockdown vs. post‐pandemic *p* = 0.0694).

**FIGURE 2:**
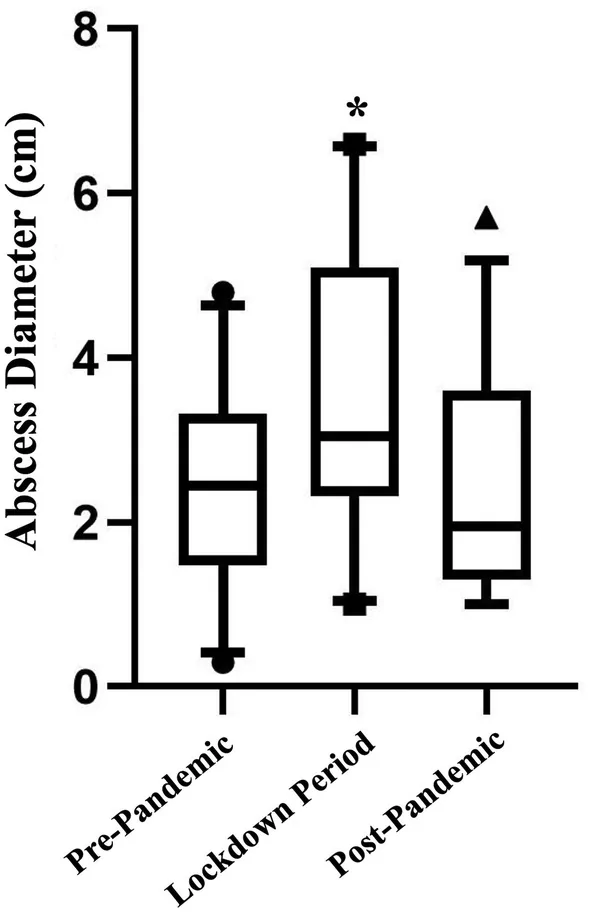
Box plot of abscess largest diameter size measured on imaging. *p < 0.05.

Regarding patient lab values, 27 (73.0%) pre‐pandemic, 17 (73.9%) lockdown patients, and 101 (75.4%) post‐pandemic patients had blood work obtained. There were no significant differences between mean WBC, ANC, ESR, or CRP levels between the three time periods (Table S2).

### 3.3 Causative Pathogen

Of the 126 patients who underwent any drainage (bedside I&D or surgical), 2 (1.6%) patients were not cultured, and 18 (14.3%) patients did not recover any organisms although 5 of these patients had a recent positive *Streptococcus pyogenes* rapid test. Of the 106 (84.1%) patients with cultures that had organisms recovered, the vast majority of culture results showed expected organisms for their respective collection sites including mixed oropharyngeal flora, *Streptococcus pneumoniae* , *Streptococcus pyogenes* , *Streptococcus anginosis* group, *Haemophilus influenzae* , *Streptococcus agalactiae* , *Fusobacterium necrophorum* , Methicillin sensitive *Staphylococcus aureus* , and Methicillin resistant *Staphylococcus aureus* . There were a few notable exceptions including a pre‐pandemic *Salmonella enterica* from an intracranial abscess, a culture negative post‐pandemic patient with a *Francisella tularensis* positive serology who responded to standard Tularemia antibiotic therapy, and a post‐pandemic patient with Histoplasmosis lymphadenopathy who likely had a secondary bacterial infection.

### 3.4 Treatment

There were no significant differences between the three time periods in days until first antibiotic, days until first effective antibiotic, days until first surgical intervention, LOS in the hospital, nor duration of infection (Table 4). Additionally, the use of inappropriate empiric antibiotics between the three groups was not significantly different with 1 pre‐pandemic patient (2.7%), 2 lockdown patients (8.7%), and 17 post‐pandemic patients (12.7%) being initially treated with inappropriate empiric antibiotics (*p* = 0.202).

**TABLE 4:** Comparison of treatment duration metrics across pre‐pandemic, lockdown, and post‐pandemic periods.

Treatment durations	Pre‐pandemic (*n* = 37)	Lockdown period (*n* = 23)	Post‐pandemic (*n* = 134)	*p*
Length of stay in days (admission date—discharge date, mean, SD)	5.2 (SD 7.4)	3.7 (SD 2.9)	3.4 (SD 3.7)	0.5589
Total infection duration in days (onset of symptoms—last day of antibiotics, mean, SD)	24.7 (SD 22.9)	22.9 (SD 18.9)	28.9 (SD 30.8)	0.6241
days of antibiotic use (mean, SD)	18.7 (SD 16.3)	14.3 (SD 5.9)	20.8 (SD 16.2)	0.1326
Days until receipt of first antibiotic (mean, SD)	4.0 (SD 3.3)	8.6 (SD 18.4)	5.3 (SD 11.6)	0.0971
Days until receipt of effective antibiotic (mean, SD)	4.0 (SD 3.3)	9.2 (SD 18.7)	7.6 (SD 26.0)	0.2024
Days until first surgical intervention (days, SD)	10.1 (SD 16.5)	11.3 (SD 20.9)	16.4 (SD 36.7)	0.5162

*Note:* Mean values with standard deviations are shown for length of stay, total infection duration, antibiotic use, time to first and effective antibiotic therapy, and time to first surgical intervention. *p* values were calculated using the Kruskal–Wallis test.

The rates of surgical intervention between the pre‐pandemic, lockdown, and post‐pandemic cohorts did not differ significantly (*p* = 0.261). Among the 37 pre‐pandemic patients, 5 (13.5%) underwent beside I&D, 15 (40.5%) required operative drainage, and 17 (45.9%) did not undergo surgical intervention. During the lockdown, 7 (30.4%) patients underwent beside I&D, 10 (43.5%) required operative drainage, and 6 (26.1%) did not undergo surgical intervention. Among the 134 post‐pandemic patients, 19 (14.2%) underwent beside I&D, 67 (50.0%) required operative drainage, and 48 (35.8%) did not undergo surgical intervention (Figure 3A). Of all patients who underwent at least one surgical intervention, the frequencies of patients who required more than one are shown in Figure 3B. While the post‐pandemic cohort has more repeat surgical interventions (15 patients with > 1 surgeries) than the pre‐pandemic cohort (1 patient with > 1 surgeries), there was also no significant association between the three groups (*p* = 0.088). Inpatient hospital mortality was 0% across all three time periods.

**FIGURE 3:**
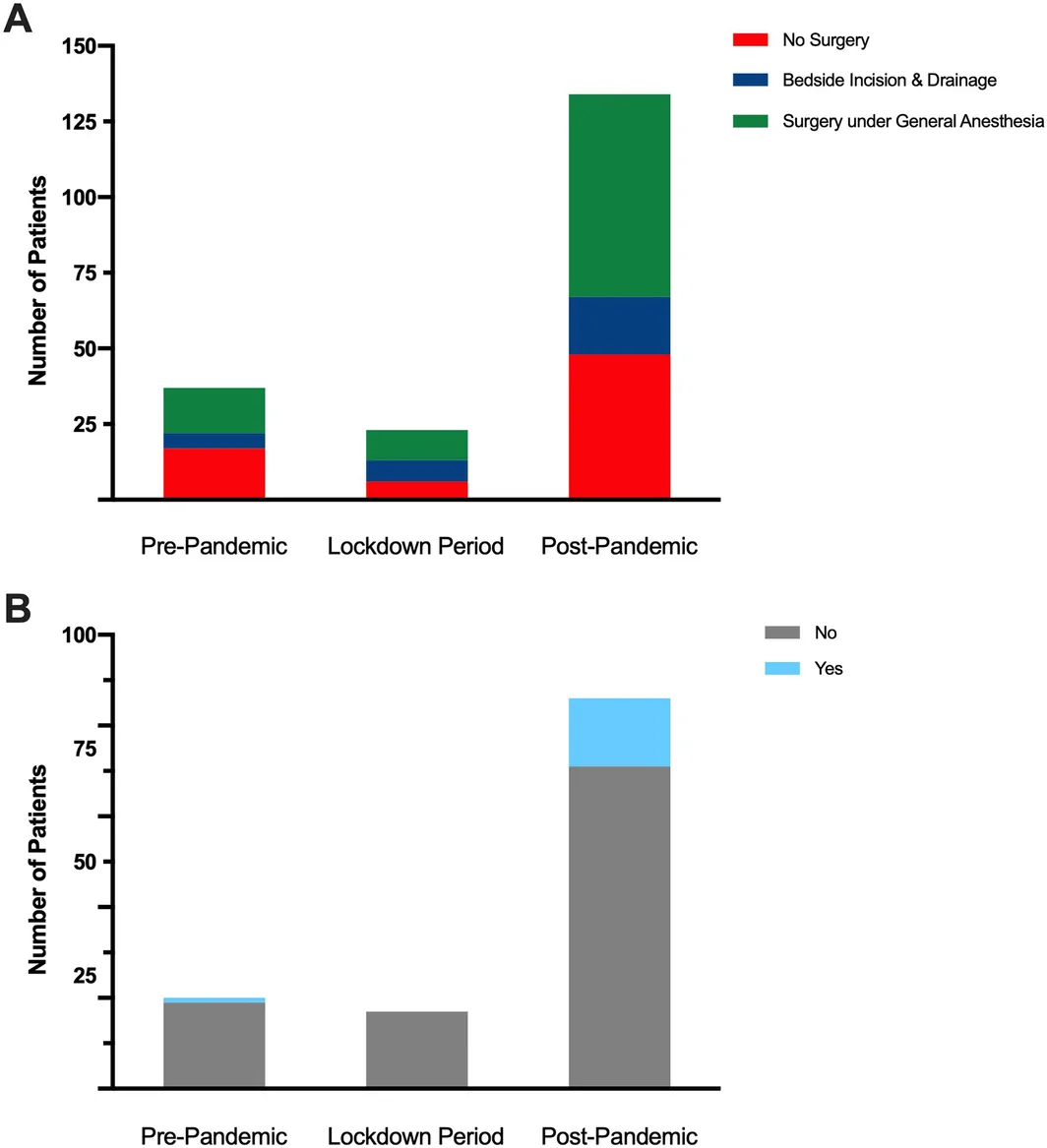
(A) Frequency of surgical management across pre‐pandemic, lockdown, and post‐pandemic periods. Patients are categorized by initial management: No surgery, bedside I&D, or surgery under general anesthesia. (B) Frequency of patients who required more than one procedure (bedside I&D or surgery under general anesthesia) was calculated. [Color figure can be viewed in the online issue, which is available at www.laryngoscope.com]

## 4 Discussion

This dual‐institution study investigated the incidence and management of pediatric head and neck abscesses across the pre‐pandemic, lockdown, and post‐pandemic time periods. Using international‐level Epic Cosmos data and chart‐reviewed institutional data, we observed a significant increase in the incidence of head and neck abscesses from the pre‐pandemic period to the lockdown period to the post‐pandemic period, with post‐pandemic incidence rates exceeding pre‐pandemic levels by more than threefold. Increased incidence occurred across nearly all anatomic subtypes and was accompanied by an increased rate of patients requiring two or more surgical interventions compared to pre‐pandemic rates. Importantly, this rise persisted despite normalization of pediatric ED volumes, supporting a true rise in disease burden rather than an artifact of healthcare utilization.

Amid the substantial rise in incidence, we observed no significant differences across time periods in patient demographics, past medical history, or available inflammatory laboratory markers. Admission rates, time to treatment, and appropriateness of empiric antibiotics also remained stable, suggesting that changes in clinical management or access to care do not fully explain the post‐pandemic increase. One notable finding was larger abscess sizes during the lockdown period compared to pre‐ and post‐pandemic cohorts, possibly reflecting early reliance on telemedicine and delayed receipt of first antibiotic initiation during the lockdown period, though this difference was not statistically significant. Microbiologic profiles were consistent with expected pathogens, including Streptococcal species, *Staphylococcus aureus* , and typical oropharyngeal commensals including anaerobes [8, 9]. We did not identify a novel dominant pathogen driving the observed increase, although this study was not powered to detect subtle shifts in organism prevalence. These findings align with national data showing increased invasive infections without a predominant causative organism [6, 7, 8, 9].

While studies outside of the United States have similarly reported a post‐pandemic increase in pediatric head and neck infections [10, 11, 12, 13, 14, 15, 16, 17], the underlying etiology remains incompletely understood and likely multifactorial. Leading immunological theories include pandemic‐induced delayed immune system priming [23, 24, 25, 26, 27], and pandemic‐related shifts in epidemiology and seasonality [28, 29, 30, 31]. Furthermore, other potential mediators, including reduced primary care access, decreased outpatient antibiotic prescribing, and socioeconomic disparities may also be contributors [32, 33, 34]. Notably, this study was not designed to assess these potential mediating factors, and the following hypotheses are discussed as possible rather than established explanations. The “immune debt” hypothesis proposes that reduced exposure to common pathogens during the lockdown period delayed normal immune maturation in children, increasing susceptibility to head and neck infection [23, 24, 25, 26, 27]. Supporting this, a large European multicenter study found significantly lower IgG levels against *Streptococcus pyogenes* and respiratory syncytial virus in 3–4 year‐old children sampled after the introduction of pandemic restrictions, consistent with an approximately 1‐year delay in immunity acquisition [26]. Furthermore, a multiplex serology study from 2020 to 2022 found that children under 5 years exhibited higher peak antibody responses following infection and more rapid antibody waning across multiple viruses compared to older patients [23]. Complementary epidemiologic studies demonstrated pandemic restrictions disrupting pathogen seasonality, resulting in atypical viral peak timing, prolonged circulation periods, and rebound surges exceeding pre‐pandemic levels for several respiratory pathogens [28, 29, 30, 31]. While these immunological mechanistic hypotheses are speculative in relation to our specific cohort, together their findings highlight the need for further investigation into age‐specific immunodynamics and the impact of pandemic‐related disruptions on immune priming during critical developmental windows. Additional United States‐based studies are needed to evaluate the role of immune debt, healthcare access, antibiotic prescribing, and socioeconomic factors in the post‐pandemic rise in pediatric bacterial infections.

This threefold rise in incidence and increased repeat surgical interventions also reflects a growing economic burden on pediatric patients, families, and the healthcare system. Prior to the pandemic, pediatric deep neck space infections accounted for over $75 million in annual United States hospital charges, with surgically managed cases incurring nearly double the costs of medically managed cases ($28,969 vs. $17,022 per patient) [35]. Given the tripled incidence we observed in this study, the current economic burden following this post‐pandemic increased incidence in head neck abscesses likely exceeds these pre‐pandemic costs. Repeat surgeries further amplify costs through prolonged hospitalizations and additional resource utilization [13, 36]. Early surgical intervention of pediatric deep space neck infections has been shown to significantly reduce LOS [37]. Beyond direct hospital charges, the post‐pandemic increase reflects broader healthcare system strain, including potentially avoidable costs from multiple imaging modalities and pre‐admission antibiotic use [38]. The convergence of higher case volumes and increased surgical intervention rates underscores the continued need to strive for improved diagnostic and treatment protocols to curb escalating financial impact.

These findings should be interpreted in the context of our study's limitations. Both institutions assessed are located within Eastern Nebraska, narrowing racial and ethnic diversity and limiting the generalizability of our findings to other geographic regions or healthcare systems. While the pathogen analysis revealed trends in microbial patterns, it was limited by incomplete culture and antibiotic sensitivity data, highlighting the need for future studies with confirmed cultures and full susceptibility testing. Additionally, the lockdown period cohort was heterogeneous in that stricter social restrictions earlier in the period evolved over time and may differ in other regions. Although our chart review confirmed true abscess diagnoses, the Epic Cosmos data lacks clinical granularity and likely would include misclassifications. The relatively small pre‐pandemic and lockdown cohorts reduced statistical power and increased the risk of Type II error, such that some clinically meaningful differences, including the higher rate of repeat surgical interventions observed post‐pandemic, may not have reached statistical significance and should not be interpreted as evidence of no effect. Supplemental patient outcomes of airway compromise and ICU admission rates were not systematically collected, limiting our ability to fully evaluate changes in disease severity and clinical outcomes across time periods. Finally, despite multiple proposed theories to explain this post‐pandemic increased incidence in pediatric head and neck abscesses, causality cannot be established, and further studies are necessary to provide a better explanation for these findings.

## 5 Conclusion

This study demonstrates a significant increase in pediatric head and neck abscess incidence following the COVID‐19 pandemic, independent of patient demographic shifts, immunodeficiency status, or treatment delays. These findings highlight the need for ongoing surveillance with prompt recognition and management of pediatric infections in the post‐pandemic era, as well as a deeper understanding of the unintended long‐term consequences of public health interventions on pediatric immune health and infectious disease patterns.

## Funding

All phases of this study were supported by the Department of Otolaryngology, University of Nebraska Medical Center.

## Conflicts of Interest

The authors declare no conflicts of interest.

## Supporting information


**Table S1:** Past medical history and clinical presentation frequencies of the pre‐pandemic, lockdown, and post‐pandemic periods. *p* values were calculated using Fisher's exact test.

**Table S2:** Laboratory values between the pre‐pandemic, lockdown period, and post‐pandemic timeframes. *p* values were calculated using the Kruskal–Wallis test.

## Data Availability

The data that support the findings of this study are available on request from the corresponding author. The data are not publicly available due to privacy or ethical restrictions.
