# Supplementary material for: Increased Pediatric Head and Neck Abscesses Following the COVID‐19 Pandemic
Source: Laryngoscope. 2026 Jul 9;136(11):5062–71. doi: 10.1002/lary.70742 (PMC13669987; doi:10.1002/lary.70742)
Supplement: Supplementary file 1 [file LARY-136-5062-s001.docx]

|  | **Pre-Pandemic (n=37)** | **Pandemic (n=23)** | **Post-Pandemic (n=134)** | **P-value** |
| --- | --- | --- | --- | --- |
| **Vaccine Status (n, %)** |  |  |  |  |
| **Haemophilus influenzae type B Vaccinated** | 34 (91.9%) | 20 (87.0%) | 127 (94.8%) | 0.2514 |
| **Pneumococcal Vaccinated** | 34 (91.9%) | 21 (91.3%) | 127 (94.8%) | 0.5818 |
| **DTaP/Tdap Vaccinated** | 36 (97.3%) | 22 (95.7%) | 128 (95.5%) | >0.9999 |
| **Immunocompromised Status (n, %)** | 0 (0%) | 0 (0%) | 0 (0%) | N/A |
| **SARS-CoV-2 Infection within 60 days prior to abscess (n, %)** | N/A | 1 (4.3%) | 5 (3.7%) | >0.9999 |
| **Recent Procedure within 6 months prior to abscess (n, %)** | 4 (10.8%) | 3 (13.0%) | 9 (6.7%) | 0.4275 |
| **Examination Findings (n, %)** |  |  |  |  |
| **Trismus** | 6 (16.2%) | 5 (21.7%) | 34 (25.4%) | 0.5407 |
| **Decreased Neck Range of Motion** | 13 (35.1%) | 7 (30.4%) | 46 (34.3%) | 0.9442 |
| **Airway Obstruction** | 2 (5.4%) | 2 (8.7%) | 18 (13.4%) | 0.4075 |
| **Fever** | 24 (64.9%) | 14 (60.9%) | 85 (63.4%) | 0.9461 |
| **Vision Changes** | 3 (8.1%) | 1 (4.3%) | 11 (8.2%) | >0.9999 |
| **Inpatient Hospital Admission (n, %)** | 26 (70.3%) | 14 (60.9%) | 101 (75.4%) | 0.3068 |

**Supplemental Table 1.** Past medical history and clinical presentation frequencies of the pre-pandemic, lockdown, and post-pandemic periods. P-values calculated using Fisher’s exact test.
