# Supplementary material for: Increased Pediatric Head and Neck Abscesses Following the COVID‐19 Pandemic
Source: Laryngoscope. 2026 Jul 9;136(11):5062–71. doi: 10.1002/lary.70742 (PMC13669987; doi:10.1002/lary.70742)
Supplement: Supplementary file 2 [file LARY-136-5062-s002.docx]

|  | **Pre-Pandemic (n=37)** | **Lockdown Period (n=23)** | **Post-Pandemic (n=134)** | **P-value** |
| --- | --- | --- | --- | --- |
| **Blood Work (mean, SD)** |  |  |  |  |
| **White Blood Cell Count (cells/µL)** | 15.69x10^3^, SD 8.6 | 15.47x10^3^, SD 4.66 | 17.76x10^3^, SD 7.51 | 0.396 |
| **Absolute Neutrophil Count (cells/µL)** | 11.46x10^3^, SD 7.33 | 9.91x10^3^, SD 3.66 | 12.8x10^3^, SD 5.95 | 0.15 |
| **C-Reactive Protein (mg/dL)** | 7.39, SD 6.47 | 10.35, SD 9.59 | 9.4, SD 8.07 | 0.726 |
| **Erythrocyte Sedimentation Rate (mm/hr)** | 61, SD 36.94 | 43.5, SD 20.66 | 56.67, SD 28.98 | 0.769 |

**Supplemental Table 2.** Laboratory values between the pre-pandemic, lockdown period, and post-pandemic timeframes. P-values were calculated using the Kruskal-Wallis test.
